# High Levels of Genetic Connectivity among Populations of Yellowtail Snapper, *Ocyurus chrysurus* (Lutjanidae – Perciformes), in the Western South Atlantic Revealed through Multilocus Analysis

**DOI:** 10.1371/journal.pone.0122173

**Published:** 2015-03-13

**Authors:** Raimundo da Silva, Ivana Veneza, Iracilda Sampaio, Juliana Araripe, Horacio Schneider, Grazielle Gomes

**Affiliations:** 1 Laboratório de Genética Aplicada, Instituto de Estudos Costeiros, Campus Bragança—Universidade Federal do Pará, Bragança, Pará, Brasil; 2 Laboratório de Genética e Biologia Molecular, Instituto de Estudos Costeiros, Campus Bragança—Universidade Federal do Pará, Bragança, Pará, Brasil; Instituto de Higiene e Medicina Tropical, PORTUGAL

## Abstract

In the present study, five loci (mitochondrial and nuclear) were sequenced to determine the genetic diversity, population structure, and demographic history of populations of the yellowtail snapper, *Ocyurus chrysurus*, found along the coast of the western South Atlantic. *O*. *chrysurus* is a lutjanid species that is commonly associated with coral reefs and exhibits an ample geographic distribution, and it can therefore be considered a good model for the investigation of phylogeographic patterns and genetic connectivity in marine environments. The results reflected a marked congruence between the mitochondrial and nuclear markers as well as intense gene flow among the analyzed populations, which represent a single genetic stock along the entire coast of Brazil between the states of Pará and Espírito Santo. Our data also showed high levels of genetic diversity in the species (mainly mtDNA), as well a major historic population expansion, which most likely coincided with the sea level oscillations at the end of the Pleistocene. In addition, this species is intensively exploited by commercial fisheries, and data on the genetic structure of its populations will be essential for the development of effective conservation and management plans.

## Introduction

Preservation of the biological diversity of any ecosystem is essential for evaluation of the distribution and connectivity of its populations [1] and the factors that determine these patterns. Considering the marine environment, opportunities for isolation to occur between populations are rare [2–4]. Many marine fish species tend to present a high degree of genetic connectivity, despite being distributed over thousands of kilometers of ocean, although this is often attributed to the intense mixing of individuals during the initial phases of development [2,5,6]. In fact, genetic connectivity has often been associated with the duration of the pelagic larval phase (PLD) [7], although a number of studies have shown that there is not always a clear relationship between the duration of this phase and the genetic homogeneity of populations [8,9].

The yellowtail snapper, *Ocyurus chrysurus* (Bloch 1790), is a lutjanid fish found in tropical and subtropical coastal regions, where it is generally associated with sandy bottoms and coral reefs [10]. This species occurs in the western Atlantic between Florida (USA) and southeastern Brazil [10,11]. Similar to other lutjanid species, *O*. *chrysurus* exhibits a pelagic larval development period of approximately 30 days [12]. Following settlement of pelagic larvae, some studies indicate that the movements of the juveniles and adults of this species are somewhat limited [13,14], which may restrict gene flow among populations. A recent study [15] that included specimens from the Florida coast and the Caribbean and analyzed both mitochondrial (ND4 gene) and nuclear data (microsatellites) found that gene flow among populations was restricted and identified four distinct stocks of *O*. *chrysurus* in the region, despite not finding high levels of genetic divergence between populations. These results were attributed to a set of factors, particularly the influence of ocean currents and limitations on the movement of the post-larvae and adults [15].

Additional studies have provided evidence of the sub-structuring of yellowtail snapper stocks in the western Atlantic, including the Caribbean [16]. Vasconcellos et al. [16] analyzed populations from the coast of Brazil (Ceará, Pernambuco, Bahia, and Espírito Santo) and the Caribbean (Belize) based on morphometric data, allozymes, and sequences of mitochondrial DNA (Control Region) and identified a single Brazilian stock, revealing significant levels of genetic sub-structuring between populations from Belize and Brazil.

In spite of the economic and ecological relevance of this species as a fishery resource, Vasconcellos et al. [16] conducted the only genetic study of the Brazilian populations of *O*. *chrysurus* reported to date. Additionally, there was a large gap between the northernmost Brazilian population examined by these authors, in the state of Ceará, and Belize. In others words, the northern limit of the Brazilian stock—or how many stocks exist—remained unclear, considering the enormous extent of the northern sector of this country’s coastline. Distinct stocks display independent evolutionary dynamics and can respond in different ways to intense fishing pressure [17]. Therefore, reliable information on these stocks is essential for fishery management and the conservation of the species.

Phylogeographic research in the western Atlantic (e.g., Brazil and Caribbean) has revealed a lack of effective barriers to gene flow in some fish species, such as two demersal lutjanids, the Southern and Northern red snappers, *Lutjanus purpureus* [5] and *Lutjanus campechanus* [6], respectively. Given the evidence of population sub-structuring between the Caribbean and Brazilian coast in the yellowtail snapper and the sampling gap in the northern Brazil in previous studies (resolved through the inclusion of samples from Maranhão and Pará) [16], the present study assessed the genetic connectivity among *O*. *chrysurus* populations distributed along more than 3,000 km of the coast of the western South Atlantic in Brazil (i. g. representing most of the species distribution in the Brazilian coast) and provides robust data on the population structure, genetic variability, and demographic history of this species.

## Materials and Methods

### Sampling

**Fig 1:**
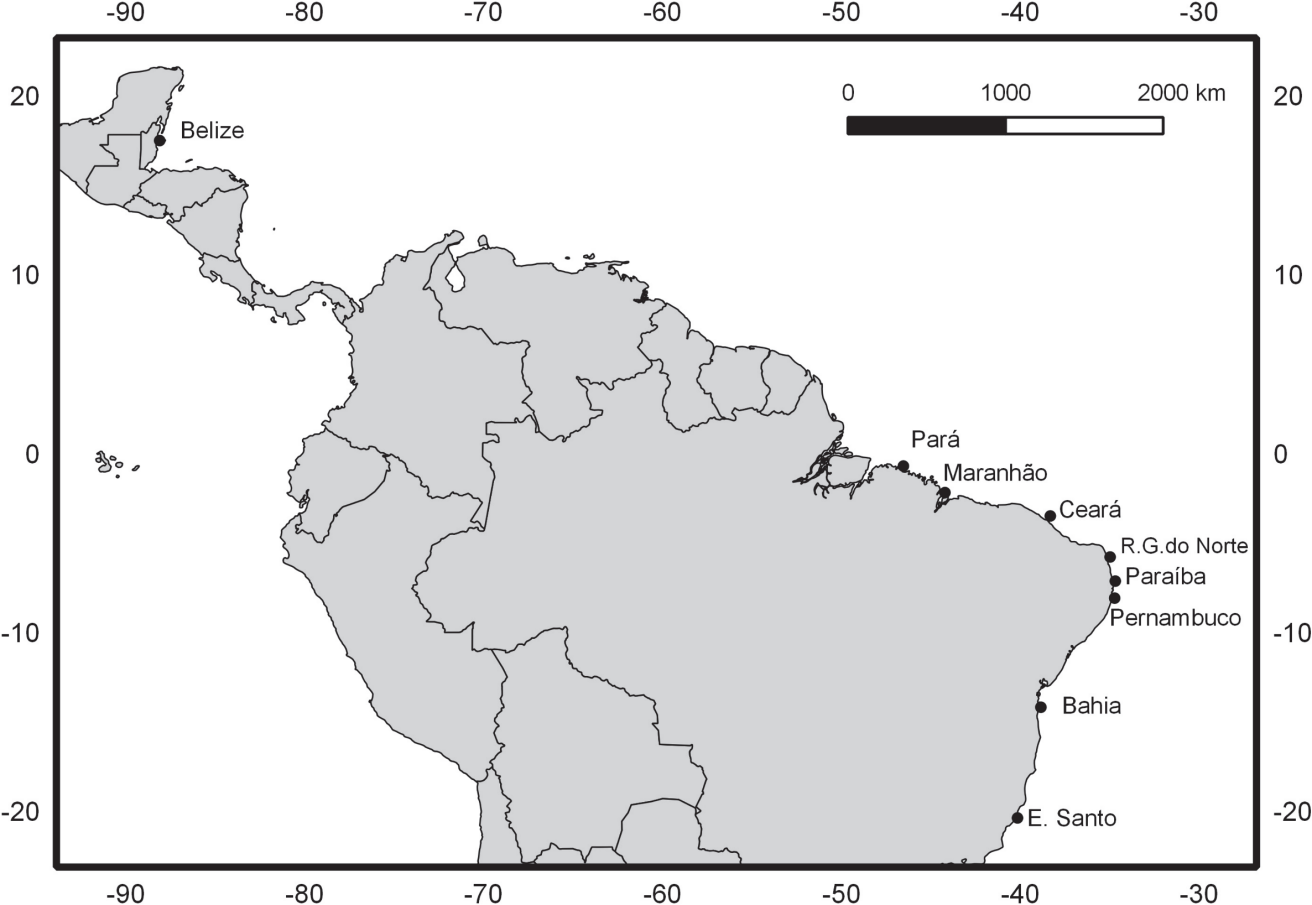
Map of collection locations for the present study. Distribution of the localities on the coast of Brazil from which the *Ocyurus chrysurus* specimens were collected for the present study, as well as location of Belize, previously sampled by Vasconcellos et al.[16].

Specimens from a total of 170 *O*. *chrysurus* adults were collected from eight localities on the coast of Brazil, between 2007 and 2012, in the states of Pará, Maranhão, Ceará, Rio Grande do Norte, Paraíba, Pernambuco, Bahia, and Espírito Santo (Fig. 1). The tissues were obtained from commercial fishing ports (obtained from the near-shore artisanal fishery) and the fishing with rafts, performed by local fisherman. The specimens were identified based on the specific literature [10,18].

### Ethics Statement

All specimens were obtained from dead individuals, procured through direct purchase from commercial landings in the localities mentioned above. *Ocyurus chrysurus* is not endangered or protected along the Brazilian Coast. Therefore, there was no need to apply for a license for collection or approval by the Animal Ethics Committee. The specimens were transported with the authorization of the Brazilian Environment Ministry.

### Laboratory procedures

In the laboratory, tissues samples (from the muscle, fin or tongue) were extracted from each specimen and frozen until analysis. All of the specimens were included in the Lutjanidae tissue bank of the Applied Genetics Laboratory at the Bragança, Campus of the Federal University of Pará (UFPA). Their genomic material was obtained using a phenol-chloroform and enzymatic extraction protocol and was precipitated with sodium acetate, isopropanol and ethanol [19].

For this study, we analyzed two mitochondrial markers, Cytochrome b (Cytb) and the control region (CR) [20,21], and three intragenic nuclear regions, Adenine Nucleotide Transporter—intron 1 (ANT-1), Growth Hormone—intron 5 (GH-5) and Insulin-Like Growth Factor 1—intron 2 (IGF-2) [22–24]. These segments were amplified using polymerase chain reaction (PCR) (Table 1). The reactions were run in a volume of 15 μL, which included approximately 100 ng of total DNA, 2.4 μL of dNTPs (1.25 mM), 1.5 μL of buffer (200 mM Tris-HCl- pH 8.0, 500 mM KCl), 0.6 μL of MgCl2 (50 mM), 0.6 μL of each primer (50 ng/μL), 0.1 μL of Taq DNA polymerase (5 U/μL), and ultrapure water added to complete the reaction volume.

**Table 1:** Primers used in the present study for Brazilian *Ocyurus chrysurus*.

Marker	Primers	Reference	Sequence (5’-3’)	Annealing
CR	Dloop-A*	Lee et al.[20]	TTCCACCTCTAACTCCCAAAGCTAG	57°C
	Dloop-G	Lee et al.[20]	CGTCGGATCCCATCTTCAGTGTTATGCTT	
Cyt b	FishCybF*	Sevilla et al. [21]	ACCACCGTTGTTATTCAACTACAAGAAC	54°C
	TrucCytbR	Sevilla et al. [21]	CCGACTTCCGGATTACAAGACCG	
IGF 2	FCmugilF*	Rodrigues-Filho [24]	GTTCACAGCGCCACACAGAC	64°C
	FCmugilR*	Rodrigues-Filho [24]	CTTGAAGGATGAATGACTATGTCCA	
GH 5	GH5F*	Hassan et al. [23]	AGGCCAATCAGGACGGAGC	57°C
	GH6R*	Hassan et al. [23]	TGCCACTGTCAGATAAGTCTCC	
ANT 1	ANTF1*	Jarman et al. [22]	TGCTTCGTNTACCCVCTKGACTTTGC	56°C
	ANTR1*	Jarman et al. [22]	CCAGACTGCATCATCATKCGRCGDC	

*Primers used for sequencing.

The amplicons were purified with PEG 8000 (polyethylene glycol) following the protocol [25] and sequenced via the dideoxy-terminal method [26] using the reagents of the Big Dye kit (ABI Prism Dye Terminator Cycle Sequencing Reading Reaction—PE Applied Biosystems, Carlsbad, CA, USA). The precipitate was sequenced through capillary electrophoresis in an ABI 3500 automatic sequencer (Applied Biosystems).

Initially, only one of the strands of each genomic region was sequenced (see Table 1). When ambiguities were observed in the chromatograms, the sample was sequenced in both directions and/or twice in the same direction (especially in the intragenic regions) to avoid errors in the identification of heterozygous individuals. For the intron of Insulin-like Growth Factor (IGF), it was necessary to design an internal primer (IGF B-5’- CATTGATATTCCTGNTCGTTCA-3’) to obtain sequences in both directions. All haplotypes were deposited in Genbank under the accession numbers KM596919 to KM507050, except for GH-5, because that fragments smaller than 200 bp are not accepted in Genbank (these sequences are listen in S1 Supporting Information).

### Database

The DNA sequences were edited and aligned in BIOEDIT v. 7.1.3.0 [27]. In the case of the nuclear loci, the individual heterozygotes were detected when double peaks were observed at the same position in both directions in the chromatograms. Heterozygotic events caused by indels were diagnosed through visual analysis of the chromatograms, with the alleles being reconstructed in INDELLIGENT v. 1.2. (http://imperialis.inhs.illinois.edu/dmitriev/indel.asp) [28].

The gametic phase of each nuclear marker was defined using the PHASE algorithm [29], available in DNAsp v 5. 10.01 [30]. The runs consisted of 1,000 burn-in iterations and 1,000 principal iterations, with a thinning interval of 1. The algorithm was applied five times, with the fifth chain being ten times longer than the others. The haplotypes that returned a probability of less than 0.8 were excluded from the analyses.

For the nuclear data, the minimum number of recombination events was estimated via the Rm method [31], available in DNAsp v 5. 10.01 [30]. As the results of this analysis are strongly affected by homoplasy [32], the significance of the number of recombination events was evaluated using the Ф_W_ test [32], available in SPLITS TREE v. 4.12.6 [33]. Linkage disequilibrium was analyzed the EM algorithm in ARLEQUIN v. 3.5.1.3 [34], which was run five times, with 20,000 permutations.

### Characterization of genetic diversity and levels of gene flow

Determination of the number of polymorphic sites and the identification of possible stop codons (in the case of codifying regions) were performed in MEGA 6 [35]. The identification, quantification, and distribution of the haplotypes were determined in DNAsp v 5. 10.01 [30]. The genetic variability of the populations was evaluated based on the haplotype (h) and nucleotide (π) diversity indices [36] obtained from ARLEQUIN v. 3.5.1.3 [34].

The haplotype network was generated using HAPLOVIEWER [37] based on a maximum parsimony tree produced in DNAPARS, available in the package PHYLIP v. 3. 6 [38], in accordance with Salzburger et al. [37].

The genetic homogeneity of the *O*. *chrysurus* populations was initially evaluated through an analysis of molecular variance (AMOVA) [39] for each marker individually and subsequently through a multilocus approach, both with 10,000 permutations. This analysis permitted partitioning of the results into within- and between-population variation. In addition, F_*st*_ values [40] were used to evaluate the gene flow between pairs of populations. These analyses were run in ARLEQUIN v. 3.5.1.3 [34], with a subsequent adjustment of the *p* values using the False Discovery Rate test [41]. To verify the existence of isolation by distance, Mantel tests were performed using a matrix of genetic (F*st*/(1-F*st*)) and geographic (km converted to Ln) distances [42]. Negative Fst values were expressed as zero. These analyses were conducted in IBDWS (http://ibdws.sdsu.edu/∼ibdws) [43], with 10,000 permutations.

For comparison between Brazilian and Caribbean populations, the control region sequences used by Vasconcellos et al. [16] (accession numbers EF624354—EF624359; EF624361—EF624373) were included in the network, AMOVA, and pairwise Fst analyses as well the Mantel test.

Bayesian methods, using STRUCTURE v. 2.3.4 [44], were applied to assign individuals to populations. This procedure places individuals into K clusters, where K is chosen in advance but can be experimentally varied throughout independent runs. K values between 1 and 8 were tested, using a model with admixture and no locprior (i. e., only genetic data is used for the assignment of individuals to a given K). For this analysis, only nuclear data were employed. Each run consisted of 1,000,000 steps (burn-in = 10%, and each value of K was implemented 10 times). The number of K was inferred by comparing the mean values of Ln Prob obtained in Structure Harvester (http://taylor0.biology.ucla.edu/structureHarvester/) [45].

Cluster analyses were also conducted in STRUCTURAMA [46]. For this analysis, the mitochondrial and nuclear data were grouped. The runs consisted of 2,000,000 generations, (burn-in = 20%). For the values of K, we employed the following distribution (K = expk (2)). The runs were summarized using the "showtogetherness" command.

To check the fit of the historical population dynamics to a model of exponential growth, we used a mismatch distribution [47] together with the SSD and raggedness indices. Mismatch analyses were conducted in DNAsp v 5 10 01 [30], rates of SSD and raggedness, were implemented in Arlequin v. 3.5.1.3 [34] based on 10, 000 permutations.

Historic fluctuations in the demography of *O*. *chrysurus* were visualized using a Bayesian Skyline Plot (BSP) [48] and an extended Bayesian Skyline Plot (EBSP) [49]. These procedures were run in BEAST v.1.7.4 [50], based on evolutionary models suggested by JMODELTEST 2.1.1 [51,52] (HKY for Cytb, IGF 2, GH 5, ANT 1 and HKY+ I + G for CR). The analyses were based on the strict molecular clock used for the teleost control region, with a substitution rate of 10% per million years [53,54].

Two runs were performed using different random seeds, including 200 million generations for each BSP run and 400 million for each EBSP run, with samples taken at intervals of 10,000 generations, 10% of which were discarded as burn-in. The convergence and mixing of the chains were inspected visually in TRACER v.1.5 [55]. The convergence and mixing were considered to be appropriate when all of the ESS values for each of the parameters analyzed were above 200.

Tajima’s *D* [56] and Fu’s *Fs* [57] were also calculated, given that in addition to the detection of possible deviations from neutrality, these values may be used to evaluate demographic patterns, such as population expansion. These analyses were run in ARLEQUIN v. 3.5.1.3 [34], with their statistical significance being assessed using 10,000 permutations.

## Results

### Mitochondrial DNA

A total of 602 base pairs (bp) was sequenced from the Control Region in 152 *O*. *chrysurus* specimens, and 645 bp of the Cytochrome b gene was sequenced in 170 specimens (Table 2). Considering the respective evolutionary rates of the two markers, similar patterns concerning their distribution and haplotype frequencies were observed in the different populations examined. In the CR, which includes 93 polymorphic sites, a total of 91 haplotypes were identified, the most common of which was shared by 27 specimens and was present at all localities except Bahia. All other CR haplotypes were either unique or occurred at low frequencies and were distinguished by a small number of mutations. Only 12 polymorphic sites were found in Cytochrome b; however, a total of 12 haplotypes were identified, two of which were very common, being shared by 74 (44%) and 67 (39%) of the specimens and being found in all of the populations analyzed.

**Table 2:** Genetic Diversity and statistics of neutrality for the Brazilian *Ocyurus chrysurus* populations analyzed in the present study.

Locus/Locality	N	Unique haplotypes	Nh	S	h (sd)	π (%)	Tajima’s *D*	Fu’s *Fs*
**Control Region**								
PA	30	16	26	50	0.983 (0.016)	1.79	−0.55 ^ns^	−12.16 **
MA	11	7	8	36	0.890 (0.091)	1.56	−1.10 ^ns^	0.10 ^ns^
CE	31	17	26	61	0.982 (0.015)	2.01	−0.79 ^ns^	−9.94 **
RN	22	11	18	50	0.956 (0.036)	1.77	−0.88 ^ns^	−5.04^(0.029)^
PB	21	7	17	51	0.966 (0.030)	1.90	−0.79 ^ns^	−3.95 ^ns^
PE	22	7	15	39	0.943 (0.035)	1.50	−0.61 ^ns^	−2.42 ^ns^
BA	8	3	6	26	0.928 (0.084)	1.93	0.83 ^ns^	1.23 ^ns^
ES	7	3	7	34	1 (0.076)	2.15	−0.38 ^ns^	−1.12 ^ns^
**Total**	**152**		**91**	**93**	**0.963 (0.010)**	**1.79**	**−1.11** ^**ns**^	**−24.22** **
**Cytochrome b**								
PA	29	1	5	5	0.615 (0.052)	0.12	−1.04 ^ns^	−1.29 ^ns^
MA	16	2	7	7	0.791 (0.076)	0.21	−1.19 ^ns^	−2.89 *
CE	31	1	6	6	0.707 (0.054)	0.18	−0.64 ^ns^	−1.15 ^ns^
RN	24	2	7	7	0.731 (0.064)	0.17	−1.23 ^ns^	−2.69^(0.027)^
PB	23	-	4	5	0.557 (0.083)	0.12	−1.22 ^ns^	−0.40 ^ns^
PE	25	1	4	4	0.616 (0.063)	0.14	−0.38 ^ns^	0 ^ns^
BA	10	-	3	2	0.711 (0.086)	0.13	0.83 ^ns^	0.25 ^ns^
ES	12	-	3	3	0.621 (0.103)	0.13	−0.04 ^ns^	0.39 ^ns^
**Total**	**170**		**12**	**12**	**0.653 (0.022)**	**0.15**	**−1.33** ^**ns**^	**−5.43** ^(**0.024)**^
**IGF 2**								
PA	22	4	12	16	0.811 (0.040)	1.43	1.95 ^ns^	0.70 ^ns^
MA	15	-	7	14	0.735 (0.054)	1.39	1.84 ^ns^	3.40 ^ns^
CE	27	4	14	14	0.808 (0.035)	1.39	2.36 ^ns^	−0.01 ^ns^
RN	21	1	11	15	0.815 (0.038)	1.44	2.25 ^ns^	1.25 ^ns^
PB	18	1	12	15	0.712 (0.069)	1.30	1.35 ^ns^	−0.28 ^ns^
PE	8	-	4	10	0.691 (0.073)	1.37	2.96 ^ns^	5.11 ^ns^
BA	8	2	8	14	0.891 (0.047)	1.37	1.08 ^ns^	0.26 ^ns^
ES	11	-	6	11	0.757 (0.074)	1.21	2.00 ^ns^	2.80 ^ns^
**Total**	**130**		**28**	**20**	**0.785 (0.017)**	**1.40**	**2.01** ^**ns**^	**−2.36** ^ns^
**GH 5**								
PA	26	-	5	4	0.451 (0.079)	0.45	−0.20^ns^	−0.66 ^ns^
MA	12	-	5	5	0.626 (0.093)	0.80	0.87 ^ns^	0.01 ^ns^
CE	33	-	4	3	0.489 (0.067)	0.52	0.89 ^ns^	0.88 ^ns^
RN	25	-	4	4	0.433 (0.079)	0.41	−0.41 ^ns^	0.06 ^ns^
PB	23	-	4	3	0.588 (0.068)	0.66	1.52 ^ns^	1.26 ^ns^
PE	13	1	5	5	0.624 (0.086)	0.84	0.36 ^ns^	0.242 ^ns^
BA	7	-	3	3	0.483 (0.142)	0.54	0.07 ^ns^	0.78 ^ns^
ES	11	-	4	3	0.463 (0.119)	0.55	0.50 ^ns^	0.05 ^ns^
**Total**	**150**		**8**	**6**	**0.510 (0.031)**	**0.57**	**0.10** ^**ns**^	**−0.96** ^**ns**^
**ANT 1**								
PA	26	-	2	1	0.110 (0.057)	0.03	−0.66 ^ns^	−0.45 ^ns^
MA	11	-	1	0	0.000 (0)	0.00	0 ^ns^	0 ^ns^
CE	33	-	2	1	0.192 (0.059)	0.06	−0.10 ^ns^	0.35 ^ns^
RN	24	-	2	1	0.119 (0.061)	0.03	−0.63 ^ns^	−0.38 ^ns^
PB	21	-	2	1	0.135 (0.068)	0.04	−0.58 ^ns^	−0.26 ^ns^
PE	7	-	2	1	0.142 (0.118)	0.04	−1.15 ^ns^	−0.59 ^ns^
BA	8	-	1	0	0.000 (0)	0.00	0 ^ns^	0 ^ns^
ES	12	-	1	0	0.000 (0)	0.00	0 ^ns^	0 ^ns^
**Total**	**142**		**2**	**1**	**0.112 (0.024)**	**0.03**	**−0.28** ^**ns**^	**−0.00007** ^**ns**^

Acronyms: N = number of specimens, Nh = number of haplotypes, S = number of segregating sites, h = haplotype diversity, π = nucleotide diversity.

*p< 0.05 (for Fs < 0.02)

**p< 0.01; ns = not significant; PA: Pará; MA: Maranhão; CE: Ceará; RN: Rio Grande do Norte; PB: Paraíba; PE: Pernambuco; BA: Bahia; Espírito Santo.

The indices of haplotypic diversity were high in the CR (h = 0.963±0.010) and lower for Cytb (h = 0.653±0.002), and the same pattern was observed in the case of nucleotide diversity, with π = 1.7% for the CR, but only π = 0.15% for Cytb (Table 2). AMOVA indicated that most of the variation in both markers occurs within populations, rather than between them, with low and non-significant Ф_ST_ values being obtained (Table 3), and this finding was further corroborated by the non-significant F_st_ values obtained in the pairwise comparisons between populations (Table 4). However, the comparison between the populations from the Brazilian coast and Belize revealed that approximately 30% of the variance is explained by differences between these two regions (Table 3). The Fst values between the Brazil and Belize populations were greater than 0.20 and were highly significant for all comparisons (Table 4), indicating particularly high differentiation between these stocks.

**Table 3:** Analysis of Molecular Variance for the Brazilian *O*. *chrysurus* populations.

C R	Variance	Variation (%)	Ф
Among groups (Brazil x Belize)	2.70579	32.92262	0.32923**
Among groups/ Within groups	−0.07523	−0.91533	−0.01365
Within of populations	5.58807	67.99271	0.32007
**CR**			
Among populations	−0.06248	−1.15973	−0.01160^ns^
Within of populations	5.44961	101.15973	
**Cyt b**			
Among populations	−0.01012	−2.08757	−0.02088 ^ns^
Within of populations	0.49468	102.08757	
**nuDNA** ^1^			
Among populations	−0.00912	−1.32	−0.01324 ^ns^
Within of populations	0.69809	101.32	

Analysis of Molecular Variance (AMOVA) for the Brazilian *O*. *chrysurus* populations analyzed in the present study, for control Region (Brazil/ Belize), Control Region (only Brazil), Cytochrome b, and nuDNA (intragenic markers –IGF 2; GH 5; ANT 1).

**- p< 0.01; ns = not significant

1 Due to the similar pattern obtained for each marker individually, we chose to show only the of the results analysis multiloci.

**Table 4:** Matrix of pairwise Fst values for the Brazilian populations of *O*. *chrysurus*.

**CR**									
	**BE**	**PA**	**MA**	**CE**	**RN**	**PB**	**PE**	**BA**	**ES**
**BE**	-								
**PA**	**0.295** **	-							
**MA**	**0.319** **	**−0.008** ^**ns**^	-						
**CE**	**0.269** **	**−0.009** ^**ns**^	**−0.014** ^**ns**^	-					
**RN**	**0.288** **	**−0.020** ^**ns**^	**−0.035** ^**ns**^	**−0.025** ^**ns**^	-				
**PB**	**0.272** **	**−0.022** ^**ns**^	**−0.014** ^**ns**^	**−0.016** ^**ns**^	**−0.025** ^**ns**^	-			
**PE**	**0.325** **	**−0.018** ^**ns**^	**−0.030** ^**ns**^	**−0.007** ^**ns**^	**−0.024** ^**ns**^	**−0.016** ^**ns**^	-		
**BA**	**0.203** **	**−0.002** ^**ns**^	**0.066** ^**ns**^	**0.001** ^**ns**^	**0.0001** ^**ns**^	**0.005** ^**ns**^	**0.035** ^**ns**^	-	
**ES**	**0.258** **	**0.014** ^**ns**^	**0.008** ^**ns**^	**0.0008** ^**ns**^	**0.009** ^**ns**^	**−0.026** ^**ns**^	**0.016** ^**ns**^	**0.077** ^**ns**^	-
**Cyt b**									
	**PA**	**MA**	**CE**	**RN**	**PB**	**PE**	**BA**	**ES**	
**PA**	-								
**MA**	**−0.023** ^**ns**^	-							
**CE**	**−0.012** ^**ns**^	**−0.030** ^**ns**^	-						
**RN**	**−0.017** ^**ns**^	**−0.030** ^**ns**^	**−0.032** ^**ns**^	-					
**PB**	**−0.008** ^**ns**^	**−0.009** ^**ns**^	**−0.021** ^**ns**^	**−0.027** ^**ns**^	-				
**PE**	**−0.015** ^**ns**^	**−0.034** ^**ns**^	**−0.006** ^**ns**^	**−0.008** ^**ns**^	**0.021** ^**ns**^	-			
**BA**	**−0.026** ^**ns**^	**−0.054** ^**ns**^	**−0.051** ^**ns**^	**−0.060** ^**ns**^	**−0.024** ^**ns**^	**−0.030** ^**ns**^	-		
**ES**	**−0.051** ^**ns**^	**−0.044** ^**ns**^	**−0.037** ^**ns**^	**−0.039** ^**ns**^	**−0.041** ^**ns**^	**−0.018** ^**ns**^	**−0.030** ^**ns**^	-	
**nuDNA**									
	**PA**	**MA**	**CE**	**RN**	**PB**	**PE**	**BA**	**ES**	
**PA**	-								
**MA**	**−0.018** ^**ns**^	-							
**CE**	**−0.015** ^**ns**^	**−0.027** ^**ns**^	-						
**RN**	**−0.015** ^**ns**^	**−0.019** ^**ns**^	**−0.015** ^**ns**^	-					
**PB**	**−0.010** ^**ns**^	**0.004** ^**ns**^	**0.003** ^**ns**^	**0.0001** ^**ns**^	-				
**PE**	**−0.030** ^**ns**^	**−0.030** ^**ns**^	**−0.017** ^**ns**^	**−0.019** ^**ns**^	**−0.023** ^**ns**^	-			
**BA**	**−0.013** ^**ns**^	**−0.034** ^**ns**^	**−0.023** ^**ns**^	**−0.023** ^**ns**^	**0.013** ^**ns**^	**−0.019** ^**ns**^	-		
**ES**	**−0.004** ^**ns**^	**−0.028** ^**ns**^	**−0.005** ^**ns**^	**−0.002** ^**ns**^	**0.047** ^**ns**^	**−0.012** ^**ns**^	−0.012*^ns^	-	

Matrix of pairwise Fst values for the Brazilian populations of *O*. *chrysurus* analyzed in the present study, for control Region, Cytochrome b, and nuDNA (intragenic markers—IGF 2; GH 5; ANT 1). Acronyms: BE- Belize, PA- Pará, MA- Maranhão, CE- Ceará, RN- Rio Grande do Norte, PB- Paraíba, PE- Pernambuco, BA- Bahia, ES- Espírito Santo

**- p = 0

*^ns^ = -p<0.05, however non-significant after FDR

^ns^- Not significant

1- Due to the similar pattern obtained for each marker individually, we chose to show only the of the results analysis multiloci.

**Fig 2:**
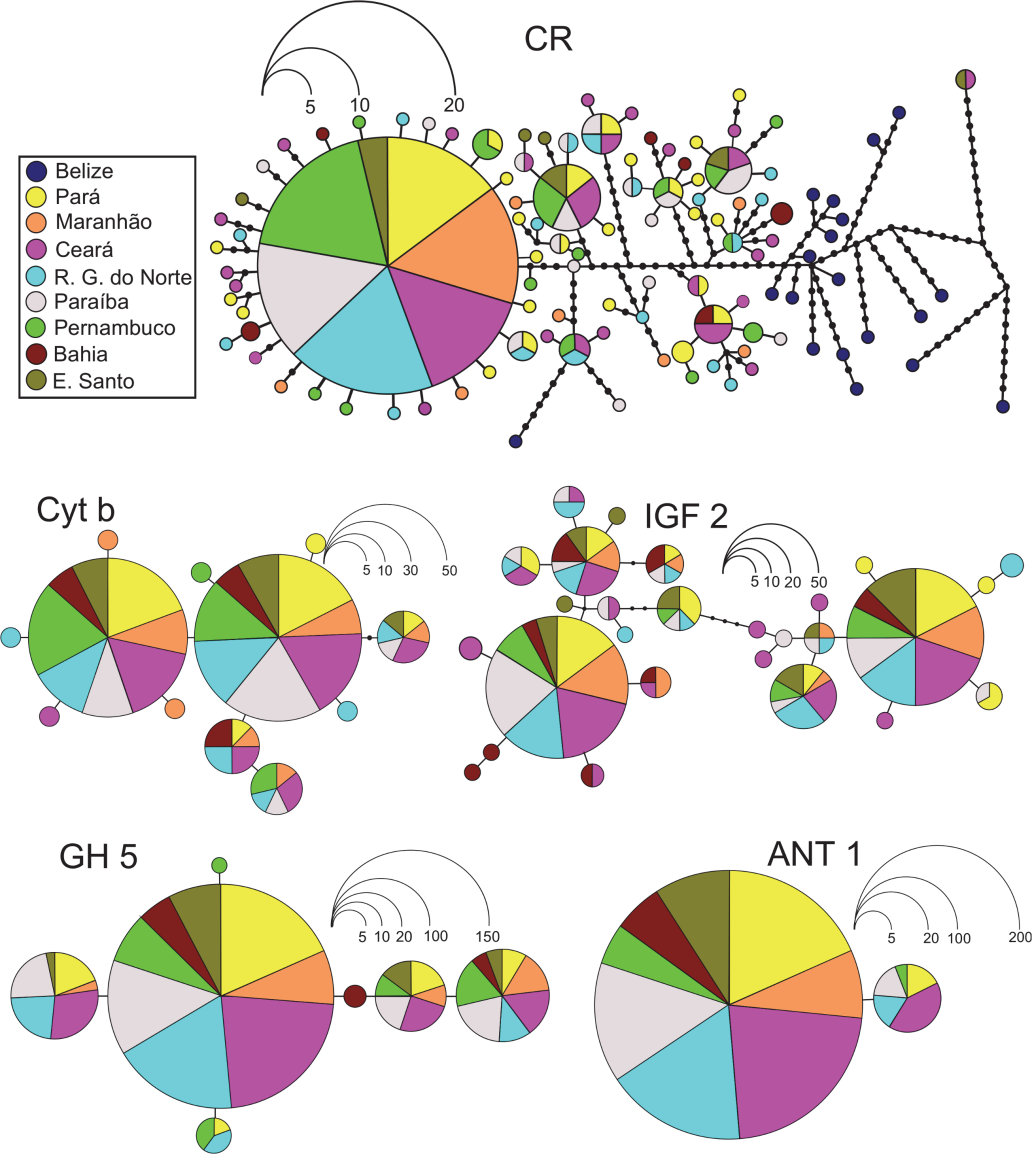
Genetic relationships among haplotypes found in the Brazilian *Ocyurus chrysurus* populations. Estimated by the maximum parsimony method, for the sequences of the Control Region, Cytochrome b, IGF 2, GH 5, and ANT. Each haplotype is represented by a circle, and the frequency of each haplotype proportional to the scale shown. Colors refer to the origin of each sample analyzed.

The genetic homogeneity of the populations from the Brazilian coast was also emphasized by the distribution of the haplotypes, given the lack of any clear geographic pattern in the network (Fig. 2). This genetic homogeneity over a broad geographic scale was further supported by the Mantel test, which rejected the scenario of isolation by distance (IBD), although when the comparisons included Belize there was some evidence in favor of a scenario with IBD, (Mantel test; r = 0.7354, p = 0.05) (S1 Fig.). The haplotypes identified in Belize were not shared by any locality analyzed along the Brazilian coast, supporting the significance of the Mantel test.

With regard to the neutrality of the data, the obtained *Fs* values were significant in some populations, or when the specimens were grouped in a single population for both mitochondrial markers (Table 2). The *D* values were not significant for any population (Table 2).

**Fig 3:**
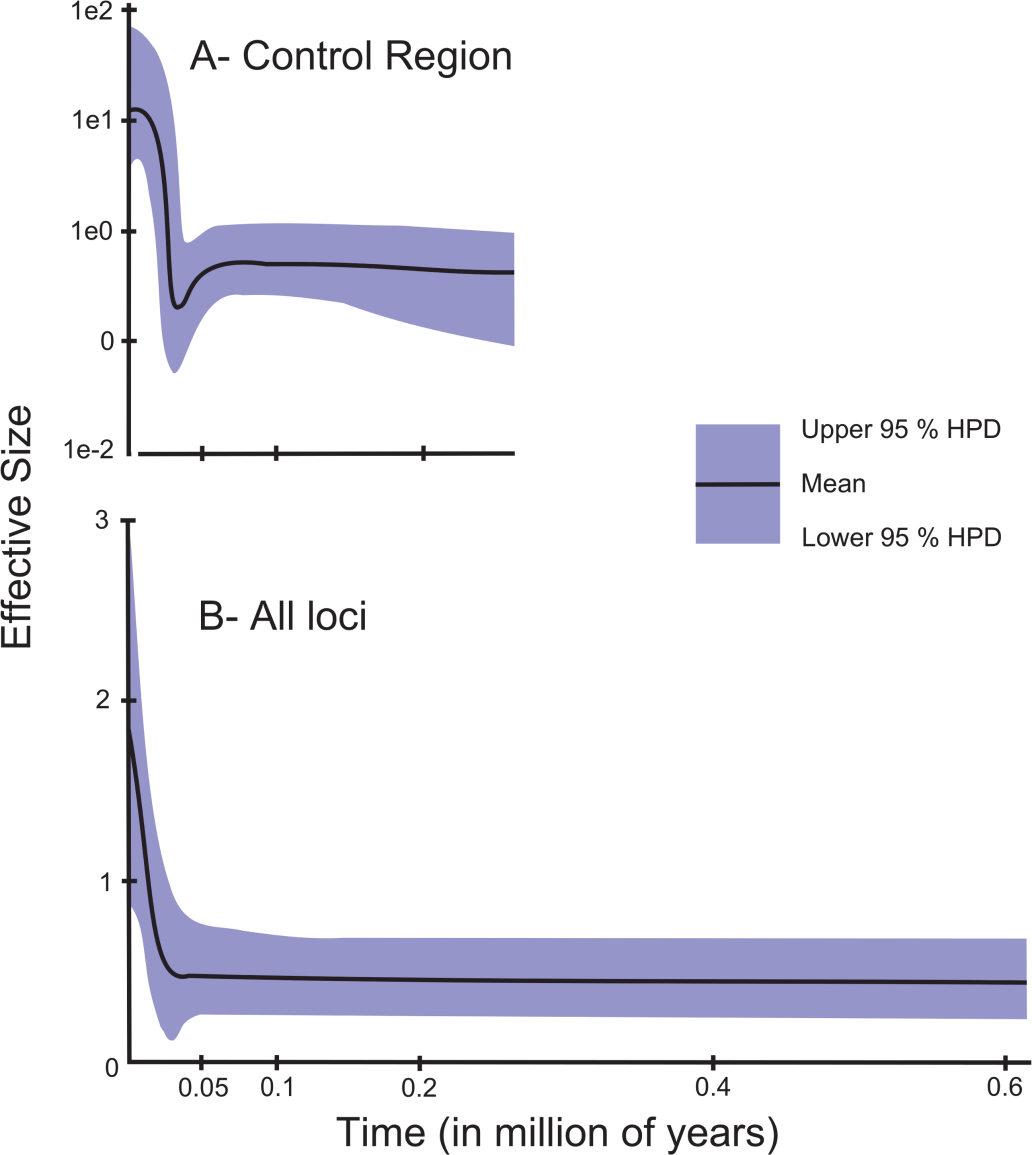
Skyline plots for Brazilian *Ocyurus chrysurus*. A. Based on the Control Region sequences (Bayesian Skyline plot). B. Extended Bayesian Skyline plot for the Brazilian *Ocyurus chrysurus* populations. Both are based on a mutation rate of 10% per million years (between lineages).

**Fig 4:**
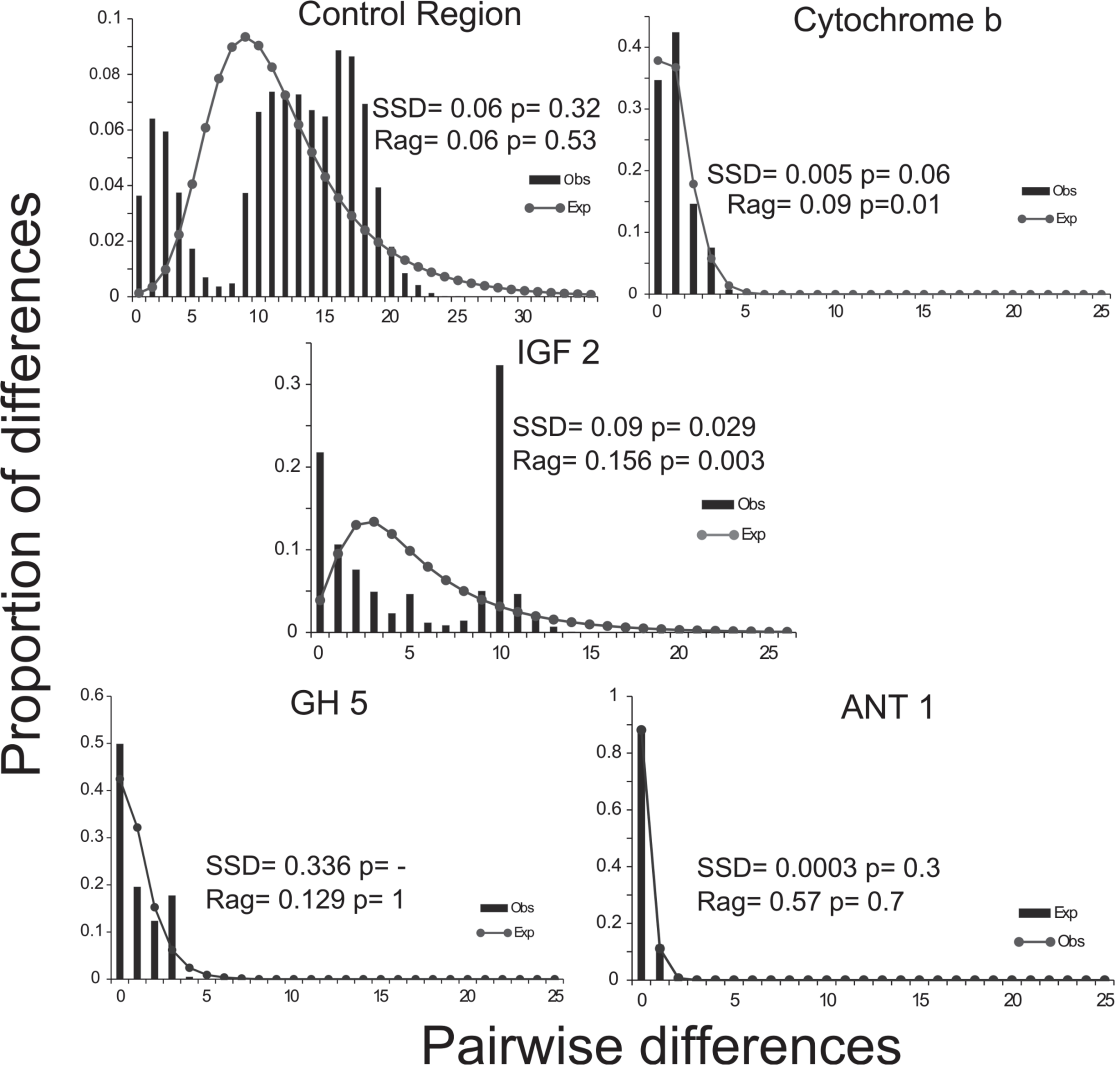
Distribution of the pairwise differences between haplotypes for *Ocyurus chrysurus* from the coast of Brazil. On the y axis the number of proportional differences is presented. X-axis represents the number of differences between pairs of sequences. Bars correspond to the observed values, and the dotted lines represent the proposed model for exponential growth.

The Bayesian Skyline Plot (Fig. 3) indicated the historic occurrence of an increase in the effective size of the *O*. *chrysurus* populations, dated to the end of the Pleistocene. These results are consistent with a process of historical expansion of yellowtail snapper populations, as indicated by significant negative *Fs* values [57] and by adjusting the mismatch distributions to model population growth (Fig. 4).

### Nuclear DNA

A fragment of 1,048 bp was sequenced for the nuclear regions, 552 bp of which corresponded to intron 2 of the Insulin-like Growth Factor (IGF) gene, which was sequenced in 130 specimens (13 individuals were excluded from the analysis due to the low *a posteriori* values recorded for their haplotypes). For this intron, an indel region of approximately 140 bp was identified. In the case of the Adenine Nucleotide Transporter (ANT) gene, 320 bp of intron 1 (together with a portion of the exons) was sequenced in 142 specimens, and 176 bp of intron 5 (together with a portion of the exon) of the Growth Hormone (GH) gene was sequenced in 150 specimens. The Фw test demonstrated the existence of recombination events only in IGF 2 (Rm = 8; p = 0.03). When the indel region was removed, recombination was not detected (p = 0. 25), and all of the analyses for this marker were performed excluding this region.

The highest diversity was recorded in the IGF 2 sequences (h = 0.785 ± 0.017; π = 1.4%), with intermediate values (h = 0.510±0.031; π = 0.57%) being recorded for GH 5 and considerably lower values being obtained for ANT 1 (h = 0.112±0.024; π = 0.03%) (Table 2).

The results of the AMOVA revealed that most of the genetic variance was present within, rather than between the populations, which is consistent with high levels of genetic similarity between populations (Table 3). This finding was reinforced by the frequency and distribution of the identified haplotypes (Fig. 2) as well as by non-significant values of the pairwise Fst (Table 4) and Mantel tests (S1 Fig.). Likewise, the Bayesian clustering analysis showed that a scenario with only one group is the most likely (S2 and S3 Figs.).

With regard to the neutrality indices (Table 2), the obtained values were negative and not significant. The mismatch distributions were unimodal for all markers, except for IGF 2, which presented a clearly bimodal distribution (Fig. 4). This pattern can be observed in populations that have experienced long intervals with stable sizes or that have undergone a subtle decrease in their effective size, followed by an event of expansion of range, or in populations who have experienced a weak bottleneck [58]. The EBSP analysis indicated the occurrence of an increase in the effective size of the *O*. *chrysurus* populations (Fig. 3), supporting the results of the BSP for the mitochondrial Control Region.

## Discussion

### Genetic structure in the yellowtail snapper

The pattern of genetic homogeneity observed in *O*. *chrysurus* in the present study is similar to that found in other lutjanids, such as *Lutjanus kasmira* [59], *Pristipomoides filamentosus* [60], *L*. *campechanus* [6], and *L*. *purpureus* [5]. All of these species exhibit a high dispersal capacity during at least one phase of their life cycle. However, a pattern of significant genetic structure has been observed for other species of this Family, such as *Lutjanus erythropterus* [53], *Lutjanus fulvus* [59], and *Lutjanus synagris* [61].

Differences in larval behavior as well as the characteristics of the environment where these species live may explain these discordant patterns of genetic connectivity and appear to directly influence the evolutionary history of these fish. However, it is important to note that *O*. *chrysurus* is not genetically homogeneous throughout the whole of its geographic distribution. Vasconcellos et al. [16] found marked differences between the Caribbean and Brazilian populations of *O*. *chrysurus*, while Saillant et al. [15] detected genetic sub-structuring in the populations of these species located in neighboring areas of the Caribbean and Gulf of Mexico. In general, these data are consistent with the existence of effective barriers in the ocean between northern Brazil and the Caribbean, (e.g., circulation patterns and ocean currents), as identified in previous phylogeographic studies [62–64], as well as between the Caribbean and the Gulf of Mexico [15,65]. Nevertheless, regarding genetic differentiation involving Brazilian populations, our results demonstrate that the model of isolation by distance cannot be discarded.

Juvenile and adult yellowtail snappers tend to remain within their area of settlement over the course of their lives [13,14]. This characteristic, in addition to specific habitat features, such as the pattern of currents, may account for the genetic differentiation observed in previous studies, with limited gene flow occurring between populations in some areas [15].

However, the larvae of *O*. *chrysurus* display a strong swimming ability [66], and they are most likely able to travel long distances, which could lead to intensive mixture of individuals. Moreover, these larvae present a planktonic phase of approximately one month and an offshore distribution, and during this larval stage, they preferably inhabit surface waters, which are more subject to the influence of ocean currents [12]. The dynamics of ocean surface circulation in the south of the western Atlantic region is primarily a result of bifurcation of the South Equatorial current [67]. Some studies, however, demonstrate that the bifurcation of the south equatorial current is not an effective barrier to dispersal for many marine taxa [68,69], furthermore, the main currents parallel to the Brazilian coast (i.e. Brazil Current / North Brazil Current) show slight seasonal changes in direction (see http://www.aoml.noaa.gov/phod/graphics/dacdata/seasonal_brazil.gif) that could allow some connectivity between the locations analyzed. Thus, all of these features are consistent with high genetic similarity along the Brazilian coast (coast Pará to Espírito Santo), as demonstrated in this study, and they may be the main factors regulating the genetic connectivity between the Brazilian populations of *O*. *chrysurus*, as observed in another lutjanid, *L*. *purpureus* [5].

### Genetic Diversity

The results of the present analysis revealed high levels of genetic variation in the investigated yellowtail snapper populations, especially in the mitochondrial markers, which presented a large number of haplotypes, similar to that recorded for *L*. *purpureus* [5] and *L*. *campechanus* [6], with similar genetic variability demonstrated between localities, over a distance of approximately 3,000 km along the Brazilian coast. This outcome would be expected for populations linked by intense gene flow, a scenario commonly observed in a number of marine species [70]. The high indices of genetic diversity recorded in the present study, especially for the mitochondrial markers, and particularly the Control Region, are a common feature of marine teleosts, including lutjanids [5,6,53,71,72], and have been recorded previously in *O*. *chrysurus* [16].

This high genetic variability cannot be interpreted as an absence of an impact of fishing on these populations because the commercial exploitation of this species is relatively recent (approximately 50 years and less than 20 generations). Examples from the literature reveal several situations where overfishing apparently shows no direct relationship with genetic diversity indices. For example, Hoarau et al. [73] did not report decreased levels of genetic variability in microsatellite markers in a temporal analysis of *Pleuronectes platissa* covering almost 100 years, even though this species has been heavily exploited since the XIX century. Additionally, Pinsky & Palumbi [74] recently performed a meta-analysis involving hundreds of fish species and observed high levels of genetic diversity for snappers of the genus *Lutjanus*, even for species that are considered to be overfished.

Distinct levels of genetic diversity were observed at the three nuclear loci analyzed in the present study. The most variable nuclear locus was the intron of the IGF 2 gene, which presented a degree of polymorphism comparable to that observed in the mitochondrial Control Region, indicating that it is a potentially useful marker for population-level and phylogeographic studies in lutjanids, as observed for *Centropomus* [75].

### Demographic History

The yellowtail snapper inhabits coastal waters [10] and is therefore relatively susceptible to sea level oscillations [76]. During the last glacial maximum, for example, approximately 90% of the present-day continental shelf of the Caribbean region was above sea level, due to a decrease in the sea level of 120 meters [77]. This situation almost certainly led to the extinction of species or lineages, as well as contraction processes and population expansion [78, 79,80].

The temperature fluctuations that occurred in this period may have had a great influence on the historical demography of a number of marine species from this region. For example, Rocha et al. [81] lists temperature fluctuations that occurred in the Atlantic (see Sachs et al. [82]) as one of the events responsible for population growth in species of the *Chromis* genus. *O*. *chrysurus* generally prefers water at temperatures between 24–30°C, with temperatures above 34°C being lethal for this species [83]. Thus, temperature changes beyond these thresholds could also lead to strong fluctuations in the effective size of *O*. *chrysurus* populations.

Historic events of population expansion are commonly identified in demographic studies on marine fish species. In the western Atlantic, events of this type have been recorded in *Cynoscion guatucupa* [84] and *Chromis multilineata* [81] as well as in lutjanids, such as the snappers *L*. *purpureus* [5] and *L*. *campechanus* [6].

The results obtained for the *O*. *chrysurus* stocks analyzed in the present study clearly indicate population expansion along the Brazilian coast. This process is similar to that observed in Caribbean *O*. *chrysurus*, although Vasconcellos et al. [16] concluded that the Brazilian populations were demographically stable because all tests neutrality (“Fs” and “D”) were unable to reject a neutral model of evolution. In this case, it is possible that the discrepancies in relation to the previous study are related to intrinsically different sensitivities of the applied neutrality tests. Finally, *Fs* test, was significant only for the total population for the mitochondrial markers examined in the present study. Fu's test shows greater statistical power and sensitivity for detecting events of geographical expansion [57] and is therefore more suitable for supporting the expansion scenario indicated by the mitochondrial markers.

The expansion of the Brazilian populations of *O*. *chrysurus* is also clear from the BSP and EBSP analyses, which identified an abrupt increase in population size approximately 25,000 years ago, which would coincide with the last glacial maximum in this region (see Barreto et al.) [85]. In fact, the recent Pleistocene glaciation events are well documented in the marine environment [82,86], and the marked changes in water temperatures and sea levels almost certainly had great effects on the historical dynamics of the population size of *O*. *chrysurus* as well as other marine organisms

### Final considerations

The present study is the most comprehensive investigation of the Brazilian populations of *Ocyurus chrysurus* undertaken to date. The results clearly revealed the existence of a single panmictic population along the Brazilian coast (*sensu* Selkoe et al.)[87], and as reported by Vasconcellos et al. [16], there is a high level of substructure between the Brazilian and Caribbean populations. However, it is worth mentioning that the absence of individuals sampled between Brazil and Belize makes it difficult to perform further analyses of the historical connectivity between populations in this geographic range.

In relation to the high levels of genetic diversity observed, mainly among mitochondrial markers, these findings cannot be interpreted as a lack of any impact of fisheries on these populations, given that the commercial exploitation of this species is relatively recent, and the markers used in the present study would not be appropriate for the detection of alterations on this time scale (see Gaither et al. [88]). Furthermore, the deterioration of genetic diversity due to overfishing is more clearly demonstrated in populations that exhibit a low effective size (e.g., a few hundred individuals) and low migration rates [74], which may not be the scenario for *Ocyurus chrysurus* along the Brazilian coast.

The data presented herein suggest that the populations of yellowtail snapper along the Brazilian coast may represent a single genetic stock, and if so, they should be managed as a unit. This conclusion has strong implications for the management of this species. For example, for various snappers, including *O*. *chrysurus*, the formation of shoals for spawning is reported to occur at a number of locations [89]. Thus, the protection of these areas should be given a higher priority because they apparently have a greater impact on the population structure over a vast area.

Further studies involving other classes of molecular markers (e.g., microsatellite markers, SNPs, adaptive loci) as well as studies tracking adults and larval dispersal should be conducted to obtain a better understanding of the structure of *O*. *chrysurus* populations.

## Supporting Information

S1 Fig Correlation between genetic distance and geographic distance between sample sites for *O*. *chrysurus*. (TIF)

S2 Fig Diagrams representing the structure of the K values (1–8). Above Mean Ln prob values ± standard deviation for each value of K. Each individual is represented by a bar. The height of the bar is proportional to the probability of the individual belonging to a given cluster.
(TIF)

S3 Fig Matrix indicating the pair-wise probability of each individual belonging to the same group. Each individual analyzed is represented by a square, and that the probability values are represented by color (see scale). Numbers: 1- Pará, 2- Maranhão, 3- Ceará, 4- R. G. do Norte, 5- Paraíba, 6- Pernambuco, 7- Bahia, 8- Espírito Santo.
(TIF)

S1 Supporting Information Codes and sequences of Growth hormone- Intron 5 utilized in the present study. (XLS)
