# Supplementary figures and images for: High Levels of Genetic Connectivity among Populations of Yellowtail Snapper, Ocyurus chrysurus (Lutjanidae – Perciformes), in the Western South Atlantic Revealed through Multilocus Analysis
Source: PLoS One. 2015 Mar 13;10(3):e0122173. doi: 10.1371/journal.pone.0122173 (PMC4359153; doi:10.1371/journal.pone.0122173)

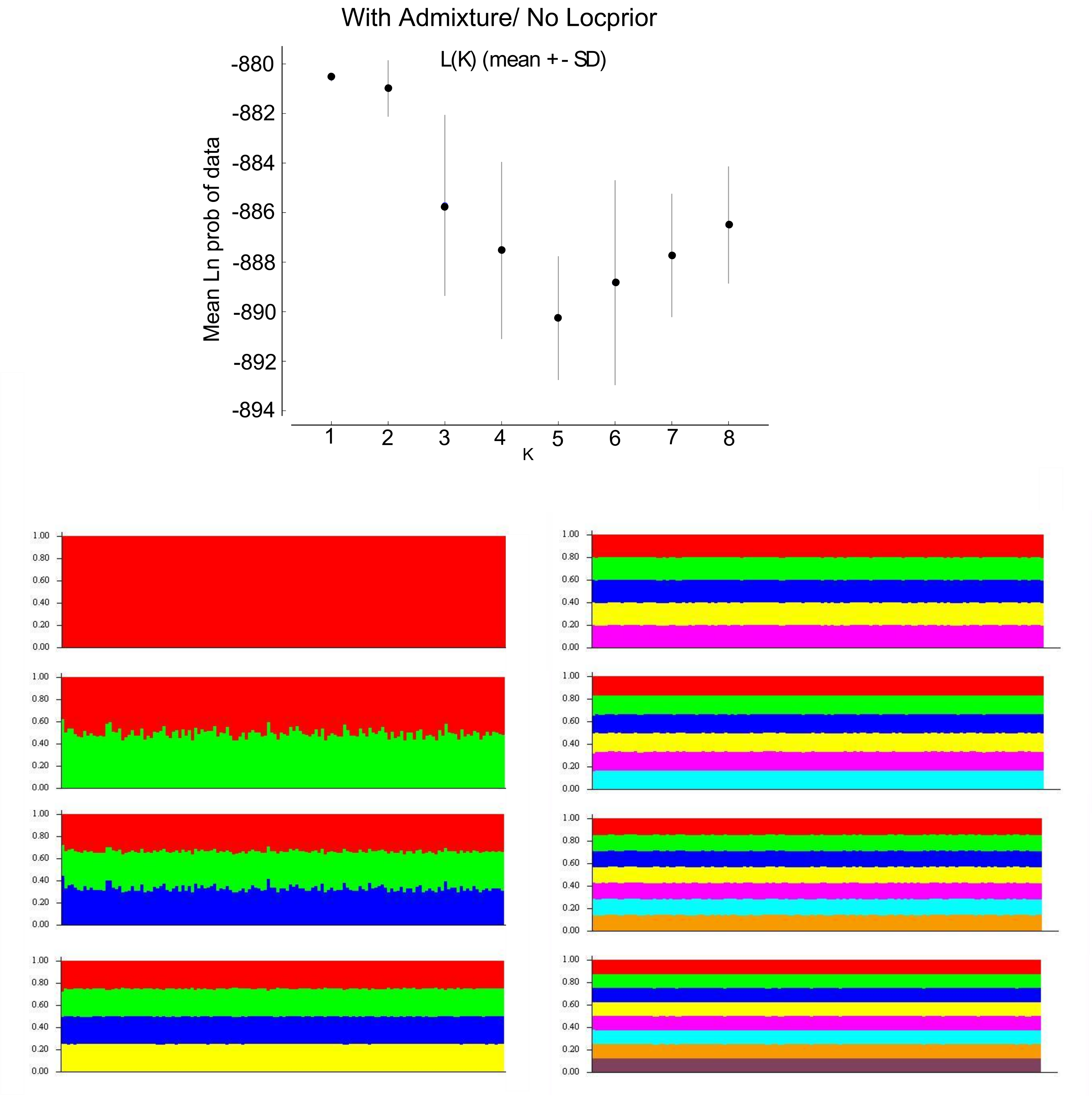

Supplement: S1 Fig — (TIF) [file pone.0122173.s001.tif]

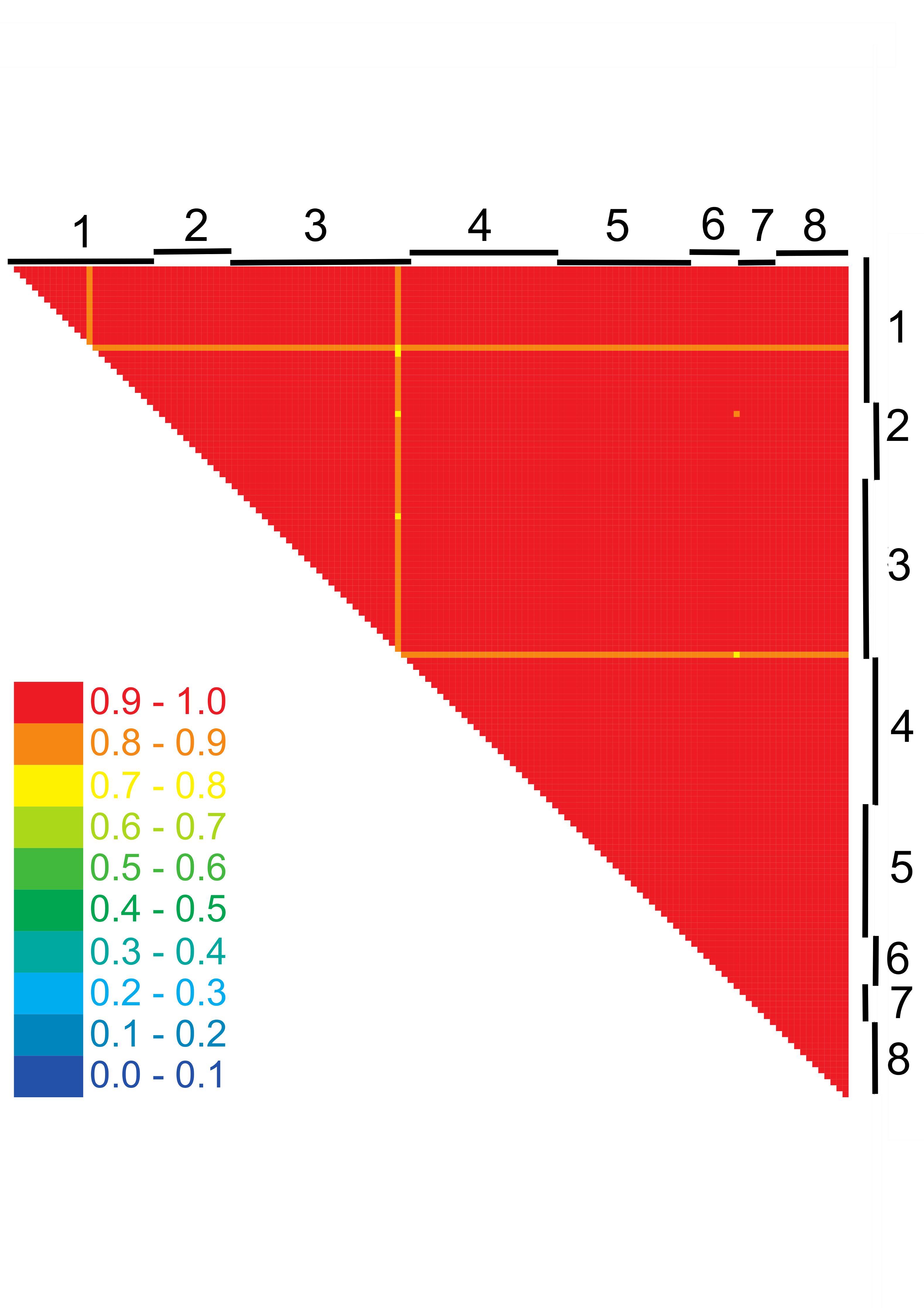

Supplement: S2 Fig — Above Mean Ln prob values ± standard deviation for each value of K. Each individual is represented by a bar. The height of the bar is proportional to the probability of the individual belonging to a given cluster. (TIF) [file pone.0122173.s002.tif]

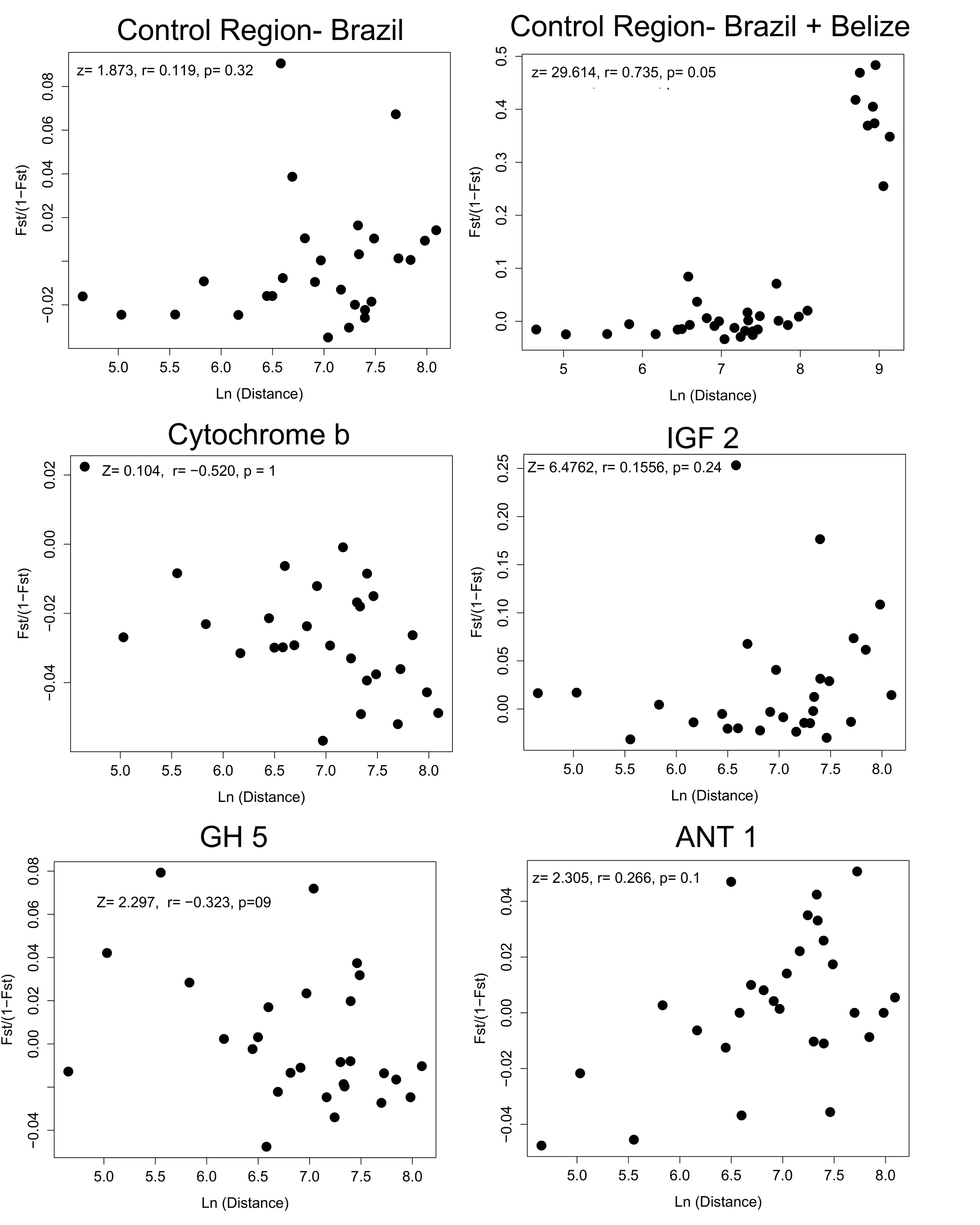

Supplement: S3 Fig — Each individual analyzed is represented by a square, and that the probability values are represented by color (see scale). Numbers: 1- Pará, 2- Maranhão, 3- Ceará, 4- R. G. do Norte, 5- Paraíba, 6- Pernambuco, 7- Bahia, 8- Espírito Santo. (TIF) [file pone.0122173.s003.TIF]
